# Whole Transcriptome Analysis of the Effects of Type **I** Diabetes on Mouse Oocytes

**DOI:** 10.1371/journal.pone.0041981

**Published:** 2012-07-24

**Authors:** Jun-Yu Ma, Mo Li, Zhao-Jia Ge, Yibo Luo, Xiang-Hong Ou, Shuhui Song, Dongmei Tian, Jin Yang, Bing Zhang, Ying-Chun Ou-Yang, Yi Hou, Zhonghua Liu, Heide Schatten, Qing-Yuan Sun

**Affiliations:** 1 State Key Laboratory of Reproductive Biology, Institute of Zoology, Chinese Academy of Sciences, Beijing, China; 2 College of Life Science, Northeast Agricultural University, Harbin, China; 3 CAS Key Laboratory of Genome Sciences and Information, Beijing Institute of Genomics, Chinese Academy of Sciences, Beijing, China; 4 Department of Veterinary Pathobiology, University of Missouri-Columbia, Columbia, Missouri, United States of America; University of Michigan Medical School, United States of America

## Abstract

In mouse ovarian follicles, granulosa cells but not oocytes take up glucose to provide the oocyte with nourishments for energy metabolism. Diabetes-induced hyperglycemia or glucose absorption inefficiency consistently causes granulosa cell apoptosis and further exerts a series of negative impacts on oocytes including reduced meiosis resumption rate, low oocyte quality and preimplantation embryo degeneration. Here we compared the transcriptome of mouse oocytes from genetically derived NOD diabetic mice or chemically induced STZ diabetic mice with that of corresponding normal mice. Differentially expressed genes were extracted from the two diabetic models. Gene set enrichment analysis showed that genes associated with metabolic and developmental processes were differentially expressed in oocytes from both models of diabetes. In addition, NOD diabetes also affected the expression of genes associated with ovulation, cell cycle progression, and preimplantation embryo development. Notably, Dnmt1 expression was significantly down-regulated, but Mbd3 expression was up-regulated in diabetic mouse oocytes. Our data not only revealed the mechanisms by which diabetes affects oocyte quality and preimplantation embryo development, but also linked epigenetic hereditary factors with metabolic disorders in germ cells.

## Introduction

It has been estimated that diabetes affects 6.4% of adults (285 million) worldwide by 2010, and this number is expected to increase to 7.7% by 2030 [1]. Diabetes not only affects the health of adults, but maternal diabetes also affects female factor fertility including oocyte maturation and ovulation, and even embryonic and fetal development [2], [3]. To investigate the effects of diabetes on oocytes, experimental animal models were utilized; streptozotocin administration induced diabetes mice (STZ mice) and genetically derived non-obese diabetic mouse strains (NOD mice) are the most commonly used models for diabetes studies [2]. Diabetes of STZ mice is the result of damage of pancreatic beta-cells induced by streptozotocin administration [4], while diabetes of NOD mice is the result of attacks on the islets by cells of the immune system [5], [6]. Both STZ and NOD diabetic mice are insulin-dependent diabetes mellitus, also known as type 1 diabetes, which induces mouse hyperglycemia and causes a series of metabolism-related disorders [2].

During growth and maturation, oocytes need to accumulate sufficient maternal components, and then need to accurately complete meiosis and ovulation before fertilization. Compared with the surrounding somatic granulosa cells, the oocyte itself displays a low ability to absorb glucose while energy substrate acquisition of oocytes strongly relies on the surrounding granulosa cells by gap junctions [7]. Granulosa cells, unlike oocytes, express a high glucose-affinity glucose transporter protein, termed SLC2A4 [solute carrier family 2 (facilitated glucose transporter), member 4)], and display insulin-sensitive features [8], indicating that insulin or insulin-like growth factor may play a role in glucose transport in granulosa cells, and may indirectly affect the oocyte’s energy metabolism. Conversely, oocytes secreting BMP15 and FGFs cooperate to stimulate the glucose metabolic processes of granulosa cells [9], indicating that a metabolic feedback loop may exist between oocytes and granulosa cells. Early reports showed that diabetes not only induced apoptosis of granulosa cells, but also reduced the meiosis resumption rate of oocytes [10]. Recently, reports focusing on the mechanisms underlying diabetes effects on oocyte quality showed that cell-cell communication was reduced between oocyte and cumulus cells in diabetic mice [11]. In addition, diabetes also induced oocyte mitochondrial dysfunction, which not only impairs the oocyte’s energy metabolism but also activates the apoptosis pathway [3], [12].

In mammalian reproduction, sperm does not play a significant role in early embryo development but contributes DNA and activates the egg [13] while materials and energy for cleavage stage embryogenesis are mainly provided by oocytes. Reports showed that during preimplantation embryo development, most embryos of NOD diabetic mice degenerated [14]. In addition, developmental delay of early embryos was also identified in both chemically induced and in genetic diabetic mice [2], [14], [15], [16].

Although the negative effects of diabetes on female fertility are well recognized, we know little about the mechanisms by which metabolic disorders affect oocyte quality and early embryo developmental potential. For that, by using STZ diabetic mice and NOD diabetic mice as models, we compared the transcriptomes of diabetic mouse oocytes with that of normal mouse oocytes.

## Methods

### Ethics Statement

This study was approved by the Animal Research Committee of the Institute of Zoology, Chinese Academy of Sciences. All animal manipulations were according to manual of Animal Research Committee. Details of animal welfare and steps taken to ameliorate suffering are included in the section about oocyte collection.

**Figure 1:**
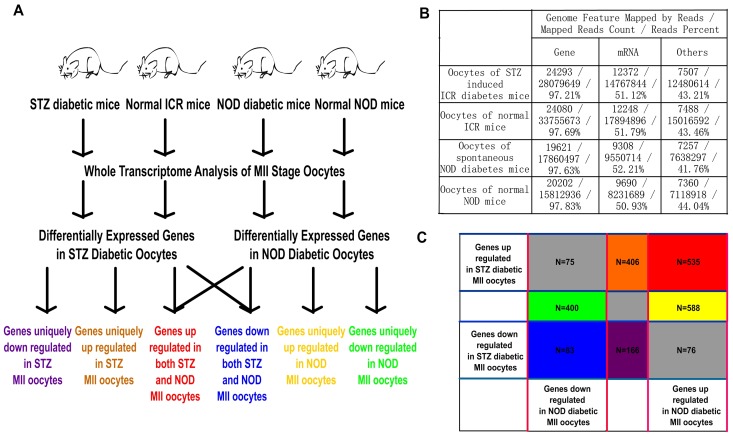
Gene expression profiles in STZ and NOD diabetic mouse models. (**A**) Schematic illustration of the experimental procedure. (**B**) Statistics of transcriptome sequencing results. (**C**) Counts of differentially expressed genes in STZ and NOD diabetic mouse MII oocytes.

### Diabetes Mouse Models and Oocyte Collection

For the construction of diabetes model mice, 8–12 weeks old mice were used, and STZ diabetic ICR strain mice were produced according the methods of Bonnevie-Nielsen [17]. The NOD spontaneous diabetic mice were purchased from Experimental Animal Facility of Nanjing University. All ICR and NOD mice with a blood glucose concentration more than 22 mM were used as diabetes model mice, and those with a blood glucose concentration less than 8 mM were used as control (5–10 mice for each group). Both diabetic and normal mice were superovulated by injecting 5U PMSG followed by 5U hCG 48 hours later. Cumulus oocyte complexes were collected 14 hours post-hCG injection, and oocytes at the metaphase of the second meiosis (MII) stage were separated from cumulus cells by hyaluronidase treatment and used for transcriptome analysis.

### SOLiD Sequencing Library Preparation and Quantitative RT-PCR Validation

The library preparation procedure mainly references the protocol of Saitou [18] and protocol provided by Applied Biosystems website (http://www.appliedbiosystems.com). The procedure is briefly summarized as follows. Each 15 oocytes were lysed in one tube and the total mRNAs were reversely transcripted to cDNAs by universe primer 1 (UP1) adaptor primers containing 24 bp oligo(dT) sequence. The remaining primers were removed and poly(A) tails to the 3′ terminal of the cDNAs were added. Universe primer 2 (UP2) primers containing 24 bp oligo(dT) were used for the synthesis of the second cDNA strand. The double strand cDNA library was amplified by UP1 and UP2 for 18 cycles, and by amine-blocked UP1 and UP2 for 14 cycles. The final amplified cDNA libraries were sent to Genome institute of Beijing and sequenced by Applied Biosystem SOLiD sequencing system. Quantitative RT-PCR was performed to evaluate the RNA sequencing results (Fig S1), primers used in RT-PCR were listed in Table S1.

### Whole Transcriptome Sequencing and Data Analysis

Total cDNAs were sequenced by SOLiD system and sequencing reads were mapped to mouse genome to extract the whole transcriptome information of oocytes. Mapped reads data were analyzed by DEGseq package [19]. Firstly, we divided the detected genes with corresponding reads count more than 10, into two groups: highly expressed genes (corresponding reads counts more than 1/10 of the mean value of all detected genes reads counts) and lowly expressed genes (reads counts less than 1/10 of the mean value). Secondly, fold change selection method was used for differentially expressed genes selection. For highly expressed genes, whose log2 (fold change) values bigger than 0.8 or less than −0.8 were selected as up-regulated genes or down-regulated genes. For lowly expressed genes, the selection standard was set at 2 to reduce the false positive rate, and genes log2 (fold change) values bigger than 2 or less than −2 were selected as up-regulated genes or down-regulated genes, respectively. Fisher exact test was used for differentially expressed genes biological processes enrichment analysis [20]. KEGG pathway graphs were created from KEGG color map website. SOLiD sequencing data was submitted to SRA with accession id: SRA037765. All R scripts can be obtained on request.

**Figure 2:**
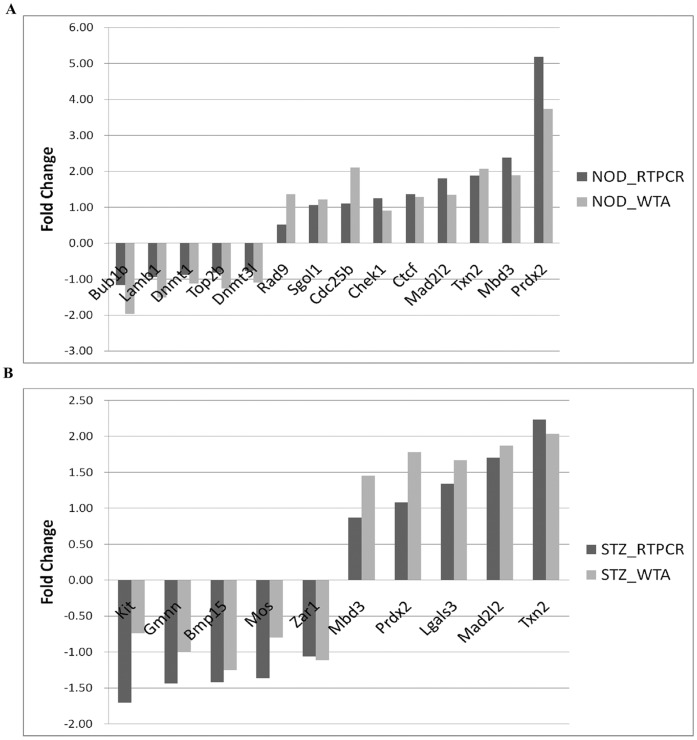
Quantitative RT-PCR validation of the transcriptome sequencing results. STZ (**A**) and NOD (**B**) diabetic MII oocytes transcriptome data were both evaluated by qRT-PCR. Correlation coefficient of the two experiment groups were 0.974 (STZ group) and 0.971 (NOD group), respectively.

## Results

### Screening Differentially Expressed Genes in Diabetic Mouse Oocytes

To compare the oocyte transcriptomes of diabetic and normal mice, STZ and NOD diabetic mice were used as diabetes models. MII stage oocytes were isolated from diabetic model mice and corresponding normal mice. Total mRNAs were extracted and used for SOLiD whole transcriptome analysis (Figure 1A). Sequenced mRNA reads were mapped to mouse genome, and the reads number mapped to each transcript was used to represent the expression level of the transcript (Figure 1B). As a result, 16457 genes were detected in all samples, in which the reads number of 8792 genes was more than 10.

By the selection standards described in the methods, we found that the mRNA levels of 1199 genes were up-regulated and 658 genes were down-regulated in NOD diabetic oocytes, whereas mRNA levels of 1016 genes were up-regulated and 325 genes were down-regulated in STZ diabetic oocytes. Among these, 535 genes’ transcription was up-regulated and 83 genes’ tanscription was down-regulated in both STZ and NOD diabetic oocytes (Figure 1C). All differentially expressed genes in STZ diabetic oocytes and NOD diabetic oocytes are listed in Dataset S1. To evaluate the quality of the sequencing method, some differentially expressed genes were re-analyzed by quantitative RT-PCR. As a result, the RNA sequencing and RT-PCR correlation values of the STZ group and NOD group were both more than 0.97 (Figure 2), which reflected the reliability of high-throughput sequencing technology.

### The Effects of Diabetes on Biological Processes of Mouse Oocytes

The effects of diabetes on oocytes from the two models were analyzed by gene set enrichment analysis. By our methods, we found that STZ-induced diabetes mainly up-regulated the transcription of genes associated with the metabolic biological processes, such as molecular metabolic process, nitrogen compound metabolic process and oxidation-reduction process. The genes whose mRNAs decreased in STZ diabetes oocytes were enriched in proliferation, development, chemical stimulus response and biological quality regulation-associated processes (Table S2).

Compared to STZ-induced diabetes, spontaneous NOD diabetes caused more severe effects on oocytes. Not only enriched in metabolic processes, up-regulated mRNAs in NOD diabetic oocytes were also enriched in processes like cell cycle, translation initiation, chromosome segregation, and ovulation cycle process (Table 1). In addition to the similar effects induced by STZ diabetes, down-regulated mRNAs in oocytes from NOD diabetic mice were also enriched in processes such as transmembrane transport, sexual reproduction, embryo implantation, cell adhesion, and cell communication (Table 2).

**Table 1:** Gene set enrichment analysis of genes up regulated in NOD diabetes mice oocytes.

GO Acc	GO Term	P-value
**GO:0044237**	cellular metabolic process	0.000
**GO:0044238**	primary metabolic process	0.000
**GO:0043170**	macromolecule metabolic process	0.000
**GO:0002253**	activation of immune response	0.000
**GO:0072376**	protein activation cascade	0.000
**GO:0044281**	small molecule metabolic process	0.000
**GO:0009056**	catabolic process	0.000
**GO:0006955**	immune response	0.001
**GO:0006807**	nitrogen compound metabolic process	0.001
**GO:0071841**	cellular component organization or biogenesis at cellular level	0.001
**GO:0006950**	response to stress	0.001
**GO:0050789**	regulation of biological process	0.001
**GO:0009058**	biosynthetic process	0.002
**GO:0016043**	cellular component organization	0.002
**GO:0045184**	establishment of protein localization	0.003
**GO:0051707**	response to other organism	0.004
**GO:0007155**	cell adhesion	0.011
**GO:0002252**	immune effector process	0.011
**GO:0048869**	cellular developmental process	0.016
**GO:0009628**	response to abiotic stimulus	0.016
**GO:0022402**	cell cycle process	0.017
**GO:0065009**	regulation of molecular function	0.019
**GO:0044085**	cellular component biogenesis	0.022
**GO:0007049**	cell cycle	0.025
**GO:0006413**	translational initiation	0.025
**GO:0007059**	chromosome segregation	0.026
**GO:0022602**	ovulation cycle process	0.026
**GO:0065008**	regulation of biological quality	0.027
**GO:0055114**	oxidation-reduction process	0.030
**GO:0051656**	establishment of organelle localization	0.038
**GO:0033002**	muscle cell proliferation	0.038
**GO:0021700**	developmental maturation	0.042
**GO:0051301**	cell division	0.046
**GO:0009607**	response to biotic stimulus	0.046
**GO:0042698**	ovulation cycle	0.048

**Table 2:** Gene set enrichment analysis of genes down regulated in NOD diabetes mice oocytes.

GO Acc	GO Term	P-value
**GO:0007275**	multicellular organismal development	0.000
**GO:0055085**	transmembrane transport	0.000
**GO:0001763**	morphogenesis of a branching structure	0.000
**GO:0048856**	anatomical structure development	0.000
**GO:0009653**	anatomical structure morphogenesis	0.001
**GO:0019953**	sexual reproduction	0.003
**GO:0006810**	transport	0.003
**GO:0019725**	cellular homeostasis	0.004
**GO:0016043**	cellular component organization	0.006
**GO:0042221**	response to chemical stimulus	0.008
**GO:0042445**	hormone metabolic process	0.009
**GO:0007389**	pattern specification process	0.009
**GO:0007566**	embryo implantation	0.014
**GO:0009790**	embryo development	0.014
**GO:0050900**	leukocyte migration	0.015
**GO:0048869**	cellular developmental process	0.015
**GO:0007155**	cell adhesion	0.016
**GO:0071841**	cellular component organization or biogenesis at cellular level	0.016
**GO:0007154**	cell communication	0.017
**GO:0051674**	localization of cell	0.019
**GO:0048870**	cell motility	0.019
**GO:0006928**	cellular component movement	0.024
**GO:0032504**	multicellular organism reproduction	0.035
**GO:0048609**	multicellular organismal reproductive process	0.035
**GO:0006950**	response to stress	0.039
**GO:0048646**	anatomical structure formation involved in morphogenesis	0.041
**GO:0035264**	multicellular organism growth	0.048

To find the common features of diabetes mouse oocytes, we obtained the gene lists of up- or down-regulated mRNAs in both diabetic models. Results showed that genes whose mRNAs up-regulated in both diabetic models were enriched in numerous biological processes including protein activation cascade, biosynthetic process, cellular metabolic process, cellular component movement, macromolecule metabolic process, primary metabolic process, and activation of immune response. Whereas down-regulated mRNAs in both diabetic models were enriched in biological processes including response to chemical stimulus, multicellular organism development, hormone metabolic process, anatomical structure development, biosynthetic process, nitrogen compound metabolic process, neurotrophin production, vesicle-mediated transport, and anatomical structure arrangement.

### The Effects of Diabetes on Cell Cycle-associated Genes in Mouse Oocytes

**Figure 3:**
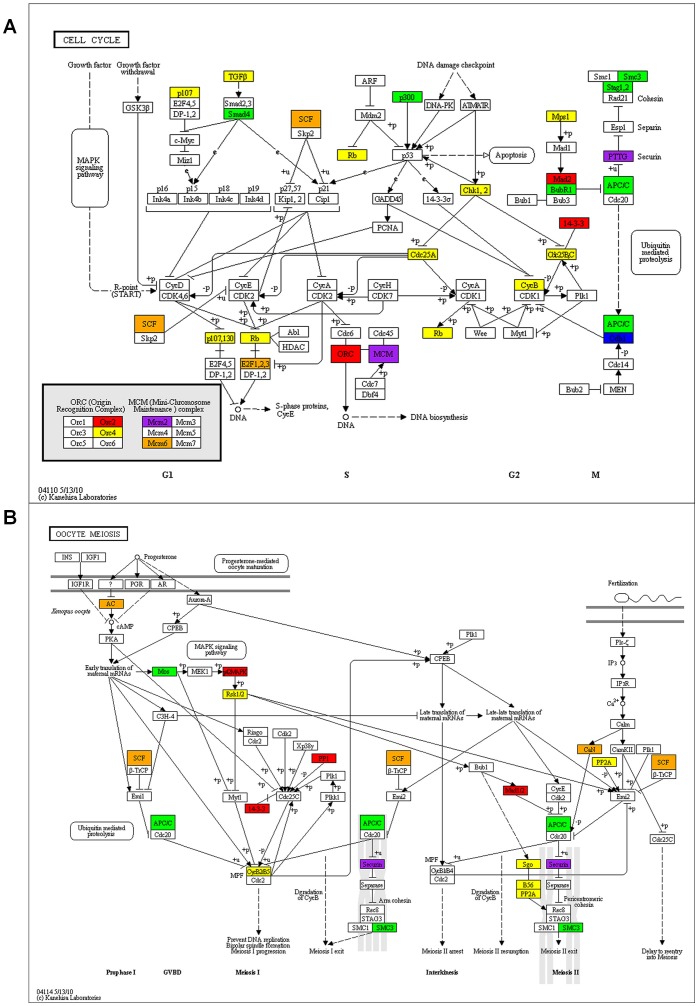
Cell cycle and meiosis KEGG pathway maps of differentially expressed genes in STZ and NOD diabetic MII oocytes. Red, genes up-regulated in both STZ and NOD diabetic mouse oocytes; Blue, genes down-regulated in both STZ and diabetic mouse oocytes; Yellow, genes up-regulated uniquely in NOD diabetic mouse oocytes; Green, genes down-regulated uniquely in NOD diabetic mouse oocytes; Orange, genes up-regulated uniquely in STZ diabetic oocytes; and Purple, genes down-regulated uniquely in STZ diabetic mouse oocytes.

To find the details on how diabetes affects meiosis progression of mouse oocytes and further post-fertilization embryo cleavage, KEGG color map was created (Figure 3). From the KEGG meiosis pathway we can see that genes like mitogen-activated protein kinase 1 (Mapk1), and MAD2 mitotic arrest deficient-like 2 (Mad2l2) were up-regulated in diabetic mouse oocytes, whereas Moloney sarcoma oncogene (Mos) and Securin (Pttg1) were down regulated. Genes differentially expressed in diabetic mouse oocytes which associated with meiosis or cell cycle processes are listed in Dataset S2.

### Cellular Localization Enrichment of Differentially Expressed Genes in both STZ and NOD Diabetic Mouse Oocytes

To find the cellular localization of differentially expressed genes in both diabetic models, cellular component enrichment was analyzed by our GO slim methods [21]. Results showed that 89 genes whose mRNA levels were significantly changed in both diabetic oocytes were localized in mitochondria (Fisher exact test p value <0.01); all cellular components which differentially expressed genes enriched in NOD and STZ mouse oocytes are listed in Table S3.

### Diabetes Affects Dnmt1 and Mbd3 Transcription in MII Oocytes

DNA methyltransferase 1 (Dnmt1), which is critical for the maintenance of DNA methylation during DNA replication [22]. The mRNA of Dnmt1 was down-regulated in both STZ and NOD diabetic MII oocytes. Genes for *de novo* DNA methylation (Dnmt3a, Dnmt3b, and Dnmt3l) expressed normally in STZ diabetic oocytes, but mRNAs of Dnmt3b and Dnmt3l were significantly down-regulated in NOD diabetic oocytes. In addition, we found that mRNAs of key methyl-CpG binding (Mbd) proteins were up-regulated in both STZ (Mbd3 and Mbd5) and NOD (Mbd2 and Mbd3) diabetic oocytes. The epigenetics-associated genes differentially expressed in two types of diabetic mouse oocytes are listed in Dataset S3.

## Discussion

### Diabetes Affects Oocyte Maturation and Oocyte Quality

Communication between oocytes and cumulus cells is critical for oocyte meiosis resumption and ovulation [23], [24], [25], [26]. Our results showed that genes significantly changed in NOD diabetic mouse oocytes were enriched in cell-cell communication, and most of which were down-regulated, indicating that diabetes weakens the communication between oocyte and cumulus cells. In addition, Bmp15 and Kit, which consisted of a negative feedback network regulating granulosa cell division [27], were down-regulated in STZ diabetic mouse oocytes, which may affect COC response to the LH, and further impair meiosis progression and oocyte ovulation [28].

Our results showed that diabetes strongly changed the transcription of cell cycle associated genes (Dataset S2). The resumption of oocyte meiosis is mainly dependent on the activity of maturation promoting factor (MPF), which includes the regulatory subunit Ccnb1 and the catalytic subunit Cdk1 [29]. In our transcriptome data, the transcription of many MPF upstream genes was significantly changed in diabetic mice oocytes. For example, fizzy/cell division cycle 20 related 1 protein (Fzr1, also known as Cdh1) is a activator of anaphase promoting complex. The Fzr1 depleted oocytes can overcome the meiosis resumption inhibition by milrinone and promote the meiosis resumption of the not fully grown oocytes (diameter, 60–69 µm). The increase of Ccnb1 in Fzr1 depleted oocytes indicates that Fzr1 is an upstream regulator of MPF [30]. The check point protein Bub1b (also known as BubR1) is also important for meiosis resumption. The Bub1b knocking down GV oocytes can partially breakthrough the 3-isobutyl-1-methylxanthine induced GV stage arrest. Bub1b knocking down can also induce the decrease of Fzr1 in GV oocytes [31]. The mRNAs levels of Fzr1 decreased in both STZ and NOD diabetic oocytes, and mRNAs of Bub1b decreased significantly in NOD diabetic oocytes and slightly decreased in STZ diabetic oocytes. These results indicated that diabetic oocytes could not strictly control the meiosis resumption which might cause the low quality of oocytes. For NOD diabetic mouse oocytes, transcriptions of some key DNA damage responding genes were changed significantly. The genes whose transcriptions decreased in NOD diabetic oocytes included the base excision repair associated genes like Polb, Smug1, and Ccno, the double strand break repair associated genes like Smarca5, H2afx, Mms221, and Shfm1, the nucleotide excision repair associated Dclrela, and the single strand break repair associated Xrcc1. On the other hand, transcriptions of some DNA damage checkpoint genes were up regulated, including Chek1, Rad9, Rad23b, Rad50, and Rad52. The DNA damage repair deficiency and higher expression of DNA damage check point genes may explain why the quality and maturation rates are lower in diabetic oocytes.

During oocyte maturation, mitochondria are important organelles for oocyte quality. Previous data have shown that maternal diabetes could cause the dysfunction of mitochondria, including mtDNA increase, narrowed intermembrane space, rupture of the outer membrane, decreased ATP yield, and induced decrease of tricarboxylic acid metabolites such as citrate, aspartate, and malate [12]. Here we found that expression of 89 mitochondria-associated genes was significantly changed in oocytes from both diabetic models, among which only four genes were down-regulated. These results may be caused by the increased number of mitochondria in diabetic oocytes. The increase in metabolic enzymes such as phosphoinositide dependent kinase 1 (Pdk1) may affect the metabolic processes during subsequent preimplantation embryo development.

### Diabetes Affects the Energy Production of Oocytes

**Figure 4:**
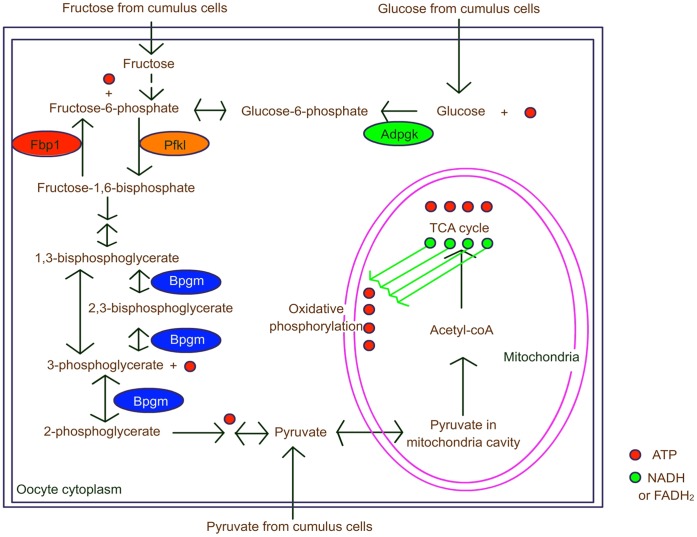
Model of diabetic mouse oocyte energy production. Red oval, genes up-regulated in both STZ and NOD diabetic mouse model oocytes; orange oval, genes up-regulated in STZ diabetic oocytes; green oval, genes down-regulated in NOD diabetic oocytes; blue oval, genes down-regulated in both STZ and NOD diabetic oocytes. Adpgk, ADP-dependent glucokinase; Fbp1, fructose bisphosphatase; Pfkl, phosphofructokinase, liver, B-type; and Bpgm, 2,3-bisphosphoglycerate mutase.

In mammalian cells, the production of ATP by glucose oxidation mainly relies on three biological processes: glycolysis in the cytoplasm; transformation of pyruvate to acetyl-coenzymeA and tricarboxylic acid (TCA) cycle in the mitochondrial matrix; and the oxidative phosphorylation process in the inner membrane of mitochondria. Both substrates of the last two processes depend on the products of glycolysis. In our results, expression level changes of enzymes associated with the phosphorylation of fructose-6-phosphate to fructose-1,6-bisphosphate and the transformation of 1,3-bisphosphoglycerate to 2-phosphoglycerate (Figure 4 and Figure S1), may retard the glycolysis process and finally, reduce the ATP production in oocytes. Evidence has been accumulated to show that important energy substrate absorption like pyruvate, fructose, and even glucose of oocytes partly relies on cumulus cells or on the environment [7], [32], [33]. Considering assisted reproductive technologies (ART), our results could provide new information on potential targets for increasing ATP content of diabetic oocytes and promote normal development of diabetic oocytes and embryos [34].

### Diabetes Affects Genes Associated with Preimplantation Embryo Development

Maternal factors are essential for early embryo development. Previous reports showed that, fewer than 20% Zar1 (zygote arrest 1) deleted embryos progressed to the 2-cell stage and none of the embryos developed to 4-cell stages [35]. Down-regulation of Zar1 transcription in diabetic mouse oocytes indicated that diabetes may affect the zygote to embryo transition. Geminin (Gmnn) gene is a cell cycle regulator which regulates the S phase to M phase transition. In our results, Gmnn was down-regulated in STZ diabetic mouse oocytes. Maternal Gmnn could not be detected until the 4-cell embryo stage, and the Gmnn deleted embryos showed developmental retardation only when the maternal Gmnn was exhausted [36]. The decrease of maternal Gmnn mRNAs may be one of the reasons to explain why preimplantation development is delayed in STZ diabetic mice.

### Epigenetic-associated Genes are Affected by Diabetes

During oocyte growth and maturation, the original imprint memory will be wiped off and oocyte-specific imprints will be reconstructed [37]. After fertilization, the zygote genome DNA is globally demethylated by DNA replication-dependent passive pathway (maternal) or DNA replication-independent active pathway (paternal) [38], [39], [40], [41]. To protect maternal and paternal imprint information from being destroyed, oocyte specific Dnmt1 (Dnmt1o) but not somatic Dnmt1, is used to maintain the methylation state of imprint-specific CpG sites in cleavage stage embryos [42], [43].

From our results, expression of Dnmt1 decreased in oocytes from both diabetes models, indicating that diabetes may threaten normal imprint of the mouse genome. In addition, some methyl-CpG binding domain proteins were up-regulated in diabetic oocytes, such as Mbd3. Evidence showed that Mbd3 could maintain imprint of paternal H19, but with no effects on other imprinted genes [44]. The change of imprint memory inheritance-associated genes in diabetic mouse oocytes showed that epigenetic markers were affected by the metabolism disorders induced by diabetes.

## Supporting Information

Figure S1 **Oxidative phosphorylation and Glycolysis/Gluconeogenesis KEGG pathway maps of differentially expressed genes in STZ and NOD diabetic MII oocytes.** Red, genes up-regulated in both STZ and NOD diabetic mouse oocytes; Blue, genes down-regulated in both STZ and diabetic mouse oocytes; Yellow, genes up-regulated uniquely in NOD diabetic mouse oocytes; Green, genes down-regulated uniquely in NOD diabetic mouse oocytes; Orange, genes up-regulated uniquely in STZ diabetic oocytes; and Purple, genes down-regulated uniquely in STZ diabetic mouse oocytes.
(PDF)

Table S1 **Samples for quantitative RT-PCR were extracted by the same methods as SOLiD sequencing library preparation.**
(DOC)

Table S2 **Gene set enrichment analysis of genes down or up regulated in STZ diabetic mice oocytes.**
(DOC)

Table S3 **Cellular components enrichment analysis of differentially expressed genes in diabetic MII oocytes.**
(DOC)

Dataset S1 **Genes Differentially Expressed in STZ and NOD diabetic Mice MII Oocytes.**
(XLS)

Dataset S2 **Expression Information of Cell Cycle Associated Genes in Diabetic Oocytes.**
(XLS)

Dataset S3 **Expression Information of Epigenetic Modification Associated Genes in Diabetic Oocytes.**
(XLS)
