# Supplementary figures and images for: Whole Transcriptome Analysis of the Effects of Type I Diabetes on Mouse Oocytes
Source: PLoS One. 2012 Jul 24;7(7):e41981. doi: 10.1371/journal.pone.0041981 (PMC3404043; doi:10.1371/journal.pone.0041981)

# A

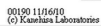

## GLYCOLYSIS / GLUCONEOGENESIS

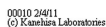

Supplement: Figure S1 — Oxidative phosphorylation and Glycolysis/Gluconeogenesis KEGG pathway maps of differentially expressed genes in STZ and NOD diabetic MII oocytes. Red, genes up-regulated in both STZ and NOD diabetic mouse oocytes; Blue, genes down-regulated in both STZ and diabetic mouse oocytes; Yellow, genes up-regulated uniquely in NOD diabetic mouse oocytes; Green, genes down-regulated uniquely in NOD diabetic mouse oocytes; Orange, genes up-regulated uniquely in STZ diabetic oocytes; and Purple, genes down-regulated uniquely in STZ diabetic mouse oocytes. (PDF) [file pone.0041981.s001.pdf]
