# Supplementary material for: Whole Transcriptome Analysis of the Effects of Type I Diabetes on Mouse Oocytes
Source: PLoS One. 2012 Jul 24;7(7):e41981. doi: 10.1371/journal.pone.0041981 (PMC3404043; doi:10.1371/journal.pone.0041981)
Supplement: Table S1 — Samples for quantitative RT-PCR were extracted by the same methods as SOLiD sequencing library preparation. (DOC) [file pone.0041981.s002.doc]

**Table S1.** Samples for quantitative RT-PCR were extracted by the same methods as SOLiD sequencing library preparation.

| **Gene Symbol** | **Primers** | |
| --- | --- | --- |
| **Kit** | Forward | GGGCTAGCCAGAGACATCAG |
|  | Reverse | AGGAGAAGAGCTCCCAGAGG |
| **Gmnn** | Forward | TTGGCAGAAGTAGCTGAGCA |
|  | Reverse | ATGTACACGGCCTAGCATCC |
| **Bmp15** | Forward | CCATTTTTGAGCTCCTCAGC |
|  | Reverse | AGAGCCCAACTGGACACATC |
| **Mos** | Forward | AGCCACTTACCACGGTGTTC |
|  | Reverse | GGTGGCACCGTAGATGACTT |
| **Zar1** | Forward | TCAAACAGTTCTGCCGAGTG |
|  | Reverse | CCATTAGCCCCATCTAGCAG |
| **Mbd3** | Forward | TGAGCAGTGACCCAACTGAG |
|  | Reverse | CCAGACCCTGGCCTGTAATA |
| **Prdx2** | Forward | AGGACTTCCGAAAGCTAGGC |
|  | Reverse | CCTGTAAGCAATGCCCTCAT |
| **Lgals3** | Forward | GATCACAATCATGGGCACAG |
|  | Reverse | AAGGGGAAGGCTGACTGTCT |
| **Mad2l2** | Forward | TGTGTGATGCTGTCCTGGAT |
|  | Reverse | GCCCTCAGCTGTTCTTATGC |
| **Txn2** | Forward | CGGACAGTACACACCACCAG |
|  | Reverse | TGGCGACCATCTTCTCTAGC |
| **Bub1b** | Forward | AGCCATGGGTATGGTGAAGA |
|  | Reverse | AAGCCAGAGGGCTAAAAAGC |
| **Lamb1** | Forward | TTGGCTCAAGCTAACAGCAA |
|  | Reverse | GGTGCTGTAAACCGCAACTT |
| **Dnmt3l** | Forward | CCTGGTGAAGAACTGCCTTC |
|  | Reverse | GCAAAGTGAGCTGCACAGAG |
| **Sgol1** | Forward | AAAAGCTTTCCAAAGGCTGA |
|  | Reverse | GACTGGCATGGAGTGAGTGA |
| **Ctcf** | Forward | TCTTGCACATGAATTGTCACAT |
|  | Reverse | CAGCCTTCAACCCCATTACA |
| **Chek1** | Forward | ACTCTCCCATCCCCAAAAGT |
|  | Reverse | TAATGGCGGAAAGCAATCAT |
| **Cdc25b** | Forward | GCTAGAGGGAAGGCCTGACT |
|  | Reverse | TGCAACAAAGGGCCTAGACT |
| **Top2b** | Forward | TGCTCCATCATTTGGTCTGA |
|  | Reverse | TGAGCCTGATGCTTTCCTTT |
| **Rad9** | Forward | CATACCCCACTTGGCTGTCT |
|  | Reverse | ATGATTGGCAGAGGGTGTTC |
