# Supplementary material for: Whole Transcriptome Analysis of the Effects of Type I Diabetes on Mouse Oocytes
Source: PLoS One. 2012 Jul 24;7(7):e41981. doi: 10.1371/journal.pone.0041981 (PMC3404043; doi:10.1371/journal.pone.0041981)
Supplement: Table S2 — Gene set enrichment analysis of genes down or up regulated in STZ diabetic mice oocytes. (DOC) [file pone.0041981.s003.doc]

**Table S2.** Gene set enrichment analysis of genes down or up regulated in STZ diabetic mice oocytes.

| **Gene classes** | **GO acc** | **GO term** | **P-value** |
| --- | --- | --- | --- |
| **Down regulated genes in STZ diabetic mice MII oocytes** | **GO:0019725** | cellular homeostasis | 0.000 |
|  | **GO:0065008** | regulation of biological quality | 0.001 |
|  | **GO:0007275** | multicellular organismal development | 0.004 |
|  | **GO:0048869** | cellular developmental process | 0.008 |
|  | **GO:0042221** | response to chemical stimulus | 0.008 |
|  | **GO:0061351** | neural precursor cell proliferation | 0.008 |
|  | **GO:0009058** | biosynthetic process | 0.015 |
|  | **GO:0048856** | anatomical structure development | 0.018 |
|  | **GO:0003008** | system process | 0.021 |
|  | **GO:0051674** | localization of cell | 0.021 |
|  | **GO:0048870** | cell motility | 0.021 |
|  | **GO:0016044** | cellular membrane organization | 0.038 |
|  | **GO:0006928** | cellular component movement | 0.041 |
|  | **GO:0045321** | leukocyte activation | 0.042 |
| **Up regulated genes in STZ diabetic mice MII oocytes** | **GO:0009058** | biosynthetic process | 0.000 |
|  | **GO:0044237** | cellular metabolic process | 0.000 |
|  | **GO:0043170** | macromolecule metabolic process | 0.001 |
|  | **GO:0044238** | primary metabolic process | 0.003 |
|  | **GO:0006950** | response to stress | 0.003 |
|  | **GO:0006955** | immune response | 0.003 |
|  | **GO:0006807** | nitrogen compound metabolic process | 0.005 |
|  | **GO:0072376** | protein activation cascade | 0.006 |
|  | **GO:0055114** | oxidation-reduction process | 0.021 |
|  | **GO:0044085** | cellular component biogenesis | 0.035 |
|  | **GO:0070661** | leukocyte proliferation | 0.038 |
|  | **GO:0044281** | small molecule metabolic process | 0.045 |
