# Supplementary material for: Whole Transcriptome Analysis of the Effects of Type I Diabetes on Mouse Oocytes
Source: PLoS One. 2012 Jul 24;7(7):e41981. doi: 10.1371/journal.pone.0041981 (PMC3404043; doi:10.1371/journal.pone.0041981)
Supplement: Table S3 — Cellular components enrichment analysis of differentially expressed genes in diabetic MII oocytes. (DOC) [file pone.0041981.s004.doc]

**Table S3.** Cellular components enrichment analysis of differentially expressed genes in diabetic MII oocytes.

| **Gene class** | **GO acc** | **GO term** | **P-value** |
| --- | --- | --- | --- |
| **Up regulated genes in NOD diabetic mice MII oocytes** | GO:0005575 | cellular_component | 3.20E-40 |
|  | GO:0005622 | intracellular | 7.04E-16 |
|  | GO:0005623 | cell | 7.56E-12 |
|  | GO:0005739 | mitochondrion | 1.21E-11 |
|  | GO:0043226 | organelle | 2.14E-11 |
|  | GO:0005737 | cytoplasm | 5.70E-11 |
|  | GO:0005634 | nucleus | 7.26E-08 |
|  | GO:0005783 | endoplasmic reticulum | 0.000354791 |
|  | GO:0005829 | cytosol | 0.001002627 |
|  | GO:0005654 | nucleoplasm | 0.001845486 |
|  | GO:0043234 | protein complex | 0.001924637 |
|  | GO:0005840 | ribosome | 0.003400373 |
|  | GO:0005856 | cytoskeleton | 0.0128444 |
|  | GO:0005886 | plasma membrane | 0.013721477 |
|  | GO:0005730 | nucleolus | 0.028857705 |
|  | GO:0005635 | nuclear envelope | 0.029597263 |
|  | GO:0005694 | chromosome | 0.034526588 |
| **Down regulated genes in NOD diabetic mice MII oocytes** | GO:0005576 | extracellular region | 2.16E-09 |
|  | GO:0005783 | endoplasmic reticulum | 8.86E-09 |
|  | GO:0005575 | cellular_component | 4.74E-08 |
|  | GO:0005623 | cell | 3.08E-06 |
|  | GO:0005794 | Golgi apparatus | 3.32E-06 |
|  | GO:0005615 | extracellular space | 5.59E-05 |
|  | GO:0005886 | plasma membrane | 0.000655394 |
|  | GO:0005634 | nucleus | 0.002079286 |
|  | GO:0016023 | cytoplasmic membrane-bounded vesicle | 0.010086698 |
|  | GO:0005578 | proteinaceous extracellular matrix | 0.013174203 |
|  | GO:0005773 | vacuole | 0.040999544 |
| **Up regulated genes in STZ diabetic mice MII oocytes** | GO:0005575 | cellular_component | 4.87E-25 |
|  | GO:0005739 | mitochondrion | 2.48E-07 |
|  | GO:0043226 | organelle | 3.99E-06 |
|  | GO:0005840 | ribosome | 4.67E-06 |
|  | GO:0005622 | intracellular | 6.22E-06 |
|  | GO:0005623 | cell | 2.99E-05 |
|  | GO:0005634 | nucleus | 0.001723843 |
|  | GO:0005737 | cytoplasm | 0.006754407 |
|  | GO:0005730 | nucleolus | 0.019709108 |
| **Down regulated genes in STZ diabetic mice MII oocytes** | GO:0005634 | nucleus | 0.006183879 |
|  | GO:0005783 | endoplasmic reticulum | 0.039738332 |
